# The Re-shaping of Bodies: A Discourse Analysis of Feminine Athleticism

**DOI:** 10.3389/fpsyg.2020.01751

**Published:** 2020-07-24

**Authors:** Sofia M. Aanesen, Runa R. G. Notøy, Henrik Berg

**Affiliations:** Centre for the Study of the Sciences and the Humanities, University of Bergen, Bergen, Norway

**Keywords:** discourse, athletic, female body, panoptic, critical, analysis

## Abstract

Slender and skinny body ideals have been associated with psychological disorders such as eating disorders. However, the tendency to promote a “healthier” and more athletic female body ideal has received minimal critical attention. This study aims at exploring the underlying conditions for such an athletic ideal through asking: How is the female athletic body constructed in the pseudonymous contemporary women’s fitness magazine, “Xrzise”? We investigated the object of inquiry through a modified version of Parker’s Foucauldian discourse analysis. We analyzed the interviews of four athletic role models in “Xrzise” and identified four discourses: “Neo-liberal discourse,” “Health expertise discourse,” “Discourse of surveillance and control” and “Discourse of emancipation.” The “Neoliberal discourse” constructs the female athletic body as something that the individual woman should strive for by appropriately managing her own resources, abilities and skills. The “Health expertise discourse” constructs the female athletic body through a homeostatic logic where the individual is responsible and healthcare experts have the mandate to intervene in order to maintain good health. The “Discourse of surveillance and control” constructs the female athletic body as an internalized panoptic stance, disciplining women to accept hegemonic beauty ideals. The “Discourse of emancipation” accentuates that the female athletic body is alleviated from a culturally rigid body image and instead improved physical performance and functionality are considered good ends. The results and discussion indicate that the female athletic body is a result of a complex nexus of different discourses associated with the powers of economy, sex differences, institutions, and ideological forces. We have advocated that magazines like “Xrzise” can have covert disciplinary effects hidden by seemingly well-intentioned motives, which can contribute to women’s objectification of their bodies.

## Introduction

Women often turn to magazines for advice on how to achieve expected ideals within a society. Body ideals are often addressed in fitness magazines by providing different strategies for bodily self-improvement. Several studies have focused on the relationship between body ideals and eating disorders (Sweeting et al., 2009, 2010; Rosenvinge and Pettersen, 2015). Compared to other forms of media usage, reading fitness magazines is to a greater extent associated with symptoms of eating disorder and body dissatisfaction (Harrison, 2000). Markula (2001) has noted that women’s eating disorders have attracted increased attention by the fitness magazines themselves. Still, these magazines aim at inspiring women by portraying narrowly defined body ideals, which are related to women’s body related psychological disorders (Markula, 2001; Duncan and Klos, 2014). These ideals seem to be a part of a larger culture of perpetual self-development. Madsen has described this tendency as the eternal pursuit of the optimal self and questioned whether this is related to the concurrent increase in mental illness in the general population (Madsen, 2015).

Susan Bordo proposed that pathological conditions within a culture are not universal abnormalities, but rather characteristic expressions of that culture (Bordo, 1992). A central question related to this is what sort of mechanisms are at work when a society’s beauty standards are imposed on women and influence their self-perceptions (Duncan, 1994). Margaret Duncan argued that Foucault’s notion of “panoptic mechanisms” is useful to understand how women objectify their own bodies. Women internalize an ideal which creates self-surveillance leading women to conform to dominant feminine body ideals. Duncan claimed that women think this self-surveillance emanates from their own personal standards. Women thus blame themselves for having a difficult relationship to their bodies, rather than holding social institutions and practices responsible for idealizing an unrealistic feminine body ideal (Duncan, 1994).

Madsen and Ytre-Arne (2012) examined how narratives describing personal or professional change is a central feature of women’s magazines, and argued that these narratives about self-change has a therapeutic jargon. As long as women continue to be discontented with how they look, women’s magazines and the products they advertise will be in high-demand (Duncan, 1994). Thus, fronting unattainable beauty ideals serve the economic interest of the beauty and fitness industry (Duncan, 1994; Duncan and Klos, 2014). Markula’s (Markula, 2001) analysis of fitness magazines supports Bordo’s work (Bordo, 1992). By drawing on the medical establishment’s presupposition that the individual is the locus of illness, fitness magazines define body dissatisfaction as an individual responsibility, instead of directing attention to the societal standards. Research indicates that many women of normal weight are dissatisfied with their bodies and are trying to lose weight (Ferreira et al., 2016). A review from 2015 found that between 25 and 77% of teenage girls are dieting or are dissatisfied with their body (Rosenvinge and Pettersen, 2015). Although there has been a decline in traditional risk factors, there has been an increase in psychosocial disorders among Western youth (Sweeting et al., 2009). In particular, there is a strong correlation between school performance stress and psychosocial distress among females (Sweeting et al., 2010). This could be seen in relation to Madsen’s supposition that the pursuit of the “optimal” and “perfect” creates a state where life can always be improved, which entails a culture where most people feel inadequate (Madsen, 2015).

Some researchers argued that the female body ideal has moved toward a healthier, more muscular and athletic body shape (Gruber, 2007). Weight training among women has received increased media attention (Independent, 2017; The guardian, 2018). This might suggest a shift in main emphasis from how the body looks to how the body functions. This trend can be understood as an acknowledgment of the beauty ideal’s negative impact on women’s psychological health. In Markula’s (Markula, 2008) work on performative pedagogy of pilates, she analyzes the fitness industry as an educational field, shaped through cultural and political forces. Dominant cultural practices within a society, like pilates, shape women’s understanding of appropriate femininity (Markula, 2008). Rich (2005) explores the relevance of feminism in young women’s lives. Rich found that women constructed their lives around dynamics of gender, while understanding a narrative of gender inequality as a problem of the past. These narratives were drawing heavily on liberal individualism, and how success and failure are makings of their own choice (Rich, 2005). It seems plausible that discourses of feminism also account for changes in the female body ideals, where cultivating an athletic female body is to choose to be less concerned with how the body is evaluated through the male perspective.

Fitness magazines have been extensively criticized by feminist researchers for having paradoxical expectations for women’s bodies, advertising body ideals that no woman can measure up to (Duncan and Klos, 2014). How does an allegedly more healthy, realistic and attainable ideal for women fit into this puzzle, and how do fitness magazines deal with their own contradictory foundation? This paper aims at exploring discourses of the athletic female body, and its societal implications through a critical analysis of the Norwegian fitness magazine Xrsize. We used a Foucauldian discourse analysis, which is concerned with the socio-historical conditions of possibility for the studied object of inquiry and its societal implications (Willig, 2013).

## Materials and Methods

### Material

We chose to examine women’s fitness magazines. Women’s fitness magazines are not limited to specific types of sports or types of training, which made *Xrzise* relevant. According to Norwegian Media Business’ Association the magazine pseudonymously called *Xrzise* is the most circulated fitness magazine nationwide (Medianorway, 2020). Xrzise markets itself as a “safe authority” of healthy attitudes and aims at increasing young women’s self-esteem. For example, the magazine states that it aims to help and inspire readers and not induce feelings of guilt. In doing this, the magazine seems to lack awareness of or disguise their contribution to women’s body dissatisfaction.

Our analysis focused on the portrait interviews in the magazine. In each volume, one person is interviewed, and that person is typically portrayed on the front cover. Thus, the interviewee appears as a front figure for the magazine. All the interviewees share a common interest in exercise and diet. In the interviews, they describe how they stay in shape, what their motives are and how they succeeded with exercise in their personal life and/or career. We included the word “athletic” in the research question because it seems to reflect the content in the interviews; the texts are concerned with an active lifestyle and building bodily strength. The interviewees are presented as inspirational role models, some of them being professional athletes. We also noted that the interviewees’ physical appearance conformed to the typical Western feminine ideal by being slender and fit, but still with a feminine, not too muscular, figure.

The primary aim of the analysis is to identify present discursive tendencies and develop some analytical categories helping us understand the suspected contradictory nature of the content in the magazines. In order to make the material manageable we limited the analysis to four volumes of the magazine. Because the selected texts were very rich, and we found the recurring themes across all four volumes, we assumed that we reached data saturation. We also assumed that available discourses in the magazine are relatively stable for a 1-year period. Due to pragmatic reasons we chose four volumes from the past year. The complete text material included 4143 words.

### Analysis

The analysis was conducted as a modified version of Ian Parker’s discourse analysis (appendix A) (Parker, 1992). We conducted the steps as a reflexive dialog between the researchers, the research material and the research question. The most important analytical steps will be presented briefly.

We used *the athletic female body* as the discursive object in the text material. Initially, all references to the discursive object were identified. As it were, this included almost every single statement in the interviews. In the next step of analysis, we organized all discursive references to *the athletic female body* according to emerging themes or discursive constructions. All features such as “fitness,” “dieting” and “active lifestyle,” that appeared to be relevant to the issue of cultivating an athletic body, was included in this analytical step. Subsequently, we categorized these themes, and recategorized them as new themes emerged throughout the analysis. In the next step, we identified discourses and certain representations within these different themes or constructions. The aim was to explore which notions of truth that was played out by the discursive constructions, and which rules make these discursive constructions possible? Discourses also create specific types of self or subject positions. The following step in the analysis aimed at exploring these subject positions, which regulates and legitimizes what can be understood or said by someone (Parker, 1992). The next step in the analysis involved exploration of coherent systems of a particular body of knowledge within the text, and the phenomena and worldviews constructed by the discourse.

According to Parker, language can be used as an analytical tool to reflect about language itself as an object (Parker, 1992). This is parallel to the way a discourse can have a reflective capacity about its own content (e.g., a health expertise discourse thematizing some hypothetical negative effects). This reflexivity can be described as a discussion that’s driven by the more implicit content in a discourse. Because this reflexivity can be recognized in other texts that are part of the same discourse, we explored other texts to elaborate the more implicit content of the discourses analyzed.

In the last steps of the analysis we examined how discourses are closely connected to institutions, power, and ideology. Discourses can reproduce and support certain institutions through practices. For example, a medical discourse could position a patient’s body as a site for medical scrutiny and treatment. Thus, discourses and discursive practices mutually validate and support each other. Increased institutionalizing leads to new forms of governance of people both inside and outside the discourse. For example, a doctor within a medical discourse considers a patient hearing voices as having auditory hallucinations and that this abnormality should be medicated and treated. In a given historic context, a discourse can also have ideological effects in relation to a specific regime of truth. A religious discourse, for example, can under some historical circumstances contribute to a racist worldview (Parker, 1992).

## Results

In the following, we present central features of the analysis. For the sake of readability, we present multiple analytical steps together. First, we present different constructions of the athletic female body which emerged as different themes during the initial steps of the analysis. Then, we will continue by presenting the discourses derived from the different constructions, and discuss their implications in the larger socio-historic context.

### The Athletic Female Body Constructed as a Self-Realization Project

The athletic female body appears to be constructed as a kind of self-realization project. The athletic female body fulfills life goals, innate talents, and acquired abilities. Three of the four women in the interviews had tried to follow their great passion and made exercise or sports into a livelihood. In the following quote, it is implied that the pride the interviewee takes in what she has accomplished in her career is legitimate: “And however humble Kayla is, she is – fortunately – proud of what she has done.” Through statements like “Here, she reveals how to go through a really effective workout.” we get the impression that the interviewees reveal their everyday routines and tips, to let the reader be inspired and follow the interviewees’ example.

Some statements suggest that the interviewees have biological prerequisites to become good at sports: “And it’s not just pigments she has inherited; Amanda claims she has also got a fuming temper from Eastern Europe. In many ways this has come in handy in her training.” At the same time, they also have characteristics usually described as psychological, such as great willpower, courage and drive. This is reflected in the statement “It was my passion for exercise and a love for helping people which led me to study to become a personal trainer.” Thus, the interviewees are presented as if they differ from other women who are concerned with fitness and exercise, while at the same time acting as realistic role models. The reader is given the opportunity to be inspired and follow the interviewees’ example, without necessarily sharing their natural abilities.

### The Athletic Female Body Constructed as a Teleological Logic

In addition to including a goal of self-realization, the athletic female body is also constructed as having the potential to realize various benefits such as performing well “when it counts.” There is an expectation that investments in the project will yield returns, and that hard work will return profits qua an improved body. Exercising therefore has instrumental value, where the primary function is to improve the level of performance or to gain a specific desired goal. Afterward, it is often important to recover and rest: “After a show, I don’t even want to see a gym until after a month! I relax, I eat Asian food with my fiancé and do enjoyable outdoor activities.”

### The Athletic Female Body Constructed as Maintaining One’s Health

Exercise and physical efforts seem to come at a cost for the interviewees. Exercise can have corrosive effects on the body. These effects make it important to be precautious: “Fortunately, the dancers always have someone with them who make sure they get replenished with the right things, such as tailor-made juices and smoothies.” Thus, the discursive object seems to be constructed as an attempt to achieve a homeostatic balance between physical and mental exertion, and intake of nutrition and rest: “In the most intensive days we have actually been training for up to 16 h. It is super important that I get enough nutrients and that I sleep well.” If there is a persistent imbalance in the homeostatic account, it can lead to fatigue, injuries and illness.

Some of the measures the interviewees take in their everyday life seem to be concerned with safeguarding their mental health. This both entails to calm down mentally and withstand an unhealthy “body pressure” generally prevailing in western culture and quite predominantly within some sub-groups: “In Hollywood, it is impossible to protect yourself from the beauty pressure, but I try to have a healthy relationship to it.”

### The Athletic Female Body Constructed as Self-Control and Discipline

The athletic female body seems to be constructed as a project where self-discipline plays a crucial role: “Behind the 2 h of explosive dancing on stage is an endless amount of training and discipline.” An active lifestyle seems to concern something immersive, where exercise is part of a rigid lifestyle requiring commitment: “Eight-hour training 6 days a week, preventive training and a strict diet are all an essential part of Mona’s life.” Training is often linked to willpower, and is described as continuous hard work over a long period of time and strict rules both when it comes to exercise and diet. An indirect example of this is the following quote: “No matter how hard it’s been to get up and get out, I’ve never regretted a workout.” One interviewee acknowledges that she has other interests, but puts these aside to follow her career: “A normal life with man and child would not have been bad either, she admits.”

### The Athletic Female Body Constructed as a Dualism

The female athletic body appears to be constructed according to a dualistic logic, where body and mind are separate. These associations are evoked by statements such as “[The interviewee] herself says that her relationship to her own body is exceptionally relaxed.” and “[she has] at the age of 24 began a fitness trend that focuses as much on confidence and an improved self-image as it does on body and strength.” In the statement “It is a routine that my body has developed and which it now enjoys,” the body is described as something almost autonomous, having its own desires. At the same time, the mind seems to have the potential for some sort of superior governing role: “A shoulder injury disabled her for two and a half years, which she fortunately overcame thanks to her strong psyche.”

### The Athletic Female Body Constructed as a Counterpart to the Beauty Pressure

The athletic female body was constructed as a challenge to the established norms of how a woman’s body should look like. This is evident in the statement “I actually agree completely that there is no such thing as a bikini body!” The interviewees seem to indirectly make a distinction between the activities they are doing and what is termed as body pressure: “[she] believes (.) that there are some misconceptions in the industry about how to achieve your own health and fitness goals.” Through the statement “There are so many things that are more important than having a six pack. Life is so g^*⁣*⁣*⁣**^n short.” an interviewee conveys that strength and health are more important than beauty. This distinction between esthetics and health seems to challenge or replace a previous standard emphasizing beauty, but which has now lost its former value. The new health standard is presented as something better and more genuine: “I don’t believe in a quick fix or transient exercise trends. I believe in healthy lifestyle routines that can be maintained and help women feel happy and good in their own body – all year round!”

### Discourses, Power Relations, and Ideology

#### Neo-Liberal Discourse

According to our analysis and as the table above demonstrates (Table 1), the neo-liberal discourse is an overarching theme throughout the text material. Both the athletic female body constructed as a self-realizing project, teleological logic and self-control and discipline, seems to be made possible by a neo-liberal discourse. The statements imply a view of human nature where the individual’s right and duty to take care of herself is essential to an overall goal of self-realization. This is evident in the statement “This is my job and my passion, and I have had to find my own way to stay healthy.” The process of self-realization can materialize when the individual breaks her own records or competes against others. This can be understood as a process where the individual pushes herself to the limit to become the best version of herself. This, in turn, presupposes that the individual has the capacity to manage her own resources, abilities, and skills in a way that brings her closer to the goal of self-realization. Here, exercise is primarily conceived as something that yields return: “My favorite exercise is the squat because it is so incredibly good at strengthening the legs, which is extremely important for my sport.” Investments in the form of hard work leads to achieving results: “After much effort, she finally got the gold medal.”

**TABLE 1:** Constructions and discourses.

	**Neo-liberal discourse**	**Health expertise discourse**	**Discourse of surveillance and control**	**Discourse of emancipation**
*The athletic female body constructed as being a self-realization project*	**×**			
*The athletic female body constructed as a teleological logic*	**×**	**×**		
*The athletic female body constructed as maintaining one’s health*		**×**		
*The athletic female body constructed as self-control and discipline*	**×**	**×**	**×**	
*The athletic female body constructed as a dualism*	**×**		**×**	
*The athletic female body constructed as a counterpart to the beauty pressure*	**×**			**×**

One has, following the principles of market economy, individual responsibility for managing and investing one’s own resources in a manner that provides the greatest possible return by actualizing the athletic female body. From the statements we get the impression that the market is “free” and that everyone can get an athletic female body by making rational choices and committing to their goals.

Within a neo-liberal discourse, the interviewees are positioned as individuals who actually have self-actualized. They are often social media influencers, which different brands collaborate with to achieve their marketing objectives. In this context, the female athletic body and its accomplishments are the result of utilizing certain products, such as training methods, exercise equipment and food products. Consequently, athletes, coaches, lifestyle guides, and other “successful” individuals act as front figures and suppliers for the products that can develop the female athletic body. They also appear as role models and ideals for the rest of the population. The audience for such success stories can be understood as consumers within this discourse. Consumers can acquire an athletic body by following the examples of the front figures when it comes to lifestyle choices, as well as consuming products such as training methods and diet plans. This is expressed in the quotation “I hope that women from all over the world can follow me and feel confident that my advice is true and effective.”

The self-help literature is another example of texts that use a neo-liberal discourse. A central feature of the self-help literature is the assumption that the individual’s own choices leads to life changes. Self-development encompasses themes such as improving your self-esteem, relationships and everyday productivity. While emphasizing that people are “experts on their own lives,” they are also encouraged to seek advice from the expert authors of the self-help books (Rimke, 2000). The authors are experts due to academic knowledge or a solid track record of achieving success on the given topic.

Identification of a neo-liberal discourse in the analysis seems to be consistent with Madsen and Ytre-Arne’s examination of the therapeutic culture in Norwegian women’s magazines. Success stories in women’s magazines are meant to inspire the reader, with an underlying notion that making decisions that lead to self-development is good or right. These stories are also accompanied by sales advertising for certain products and lifestyles (Duncan, 1994).

According to Heywood (2006), a neoliberal discourse promotes the female athlete as a kind of «super person» who differs from other women. Heywood also argued that a fit body and athletic performance serves as an outward expression of transcendence of biological limitations associated with being a woman, and in this way transcending gender limitations. However, this can create a cultural anxiety because it violates the fundamental power structures and roles in society. Because of this anxiety, female athletes are constantly reminded of their femininity by a sexualized body focus or by being referred to as mannish or “butch” in the media (Heywood, 2006).

Discourses can also have ideological effects (Parker, 1992). However, Foucault avoided the term ideology and focused on specific regimes of truth and how these are maintained by discourses (Hall, 1997). From our analysis, *liberal individualism* seems to be a prominent ideology in the text. *Liberal individualism* implies that the individual is responsible for their own life situation. Thus, individuals should be blamed if they do not make rational choices that realize their idea of a good life (Hindess, 1993). This applies when *Xrzise* offers inspirational reading in the form of “expert advice” related to exercise and diet. This concept is closely related to the discourse of healthism, where health has become a moral imperative, and «healthiness» is synonymous with «goodness» (Crawford, 2006). This is evident in the quote “I look at skincare as important as eating healthy and exercising. Your skin is an organ, similar to the heart and lungs. A healthy skin is an important part of a healthy life.” Achieving a fit body has thus gone from simply being a preoccupation to a responsibility for the individual citizen and a representation of virtue in society: This is my job and my passion, and I’ve had to find my own way to stay healthy. I have spent a lot of time figuring out which food works for me.” An example of how healthism works through different institutions in society is Evans, Rich and Holroyd’s (Evans et al., 2004) research on the role of education in the etiology of eating disorders. Through their research they conclude that school culture, mediated through the action of teachers, peers and friends, contributes to perfectionism and pressure for girls and young women. One example is that the school environment presented dieting as a resource to cope with the cultural demands or dieting as a commodity in a weight-conscious culture (Evans et al., 2004). A neo-liberal discourse assumes that the realization of the body’s potential leads to a better society. Although individual well-being and self-realization seem to be the foremost interest in women’s magazines, paradoxically, psychological disorders are more prevalent than ever in the Western world (Madsen and Ytre-Arne, 2012). Thus, one could ask whether the perpetual strive for a better and improved ‘self’ in a world where everything is potentially achievable, leads to recurring failure, loneliness, and an experience of self-loss that can manifest as mental disorders. This may be particularly prevalent in the younger population, where uncertainty related to identity can pose a greater risk factor in terms of mental health (Eckersley, 2011). External sources, such as body ideals, can constitute important identity markers, and succeeding in living up to these standards becomes the most important parameter for self-worth.

#### Health Expertise Discourse

Based on the analysis, the athletic female body is constructed as teleological logic, maintaining one’s health and self-control and discipline. These constructions can be subsumed to a health expertise discourse. This is partly because the interviewees seem to be concerned with a kind of homeostatic balance with the primary aim of maintaining good health: “I should perhaps have been stricter with myself when it comes to diet, but I think food and snacks taste way too good not to eat. Since I also work out as much as I do, I do not think it is such a big deal if I binge now and then.” In this quote, individual responsibility is linked to good health. Good health, in turn, is an important prerequisite for the athletic female body: “Personally, I have rather put another, healthy pressure on myself, which is about having good enough health to be able to perform the best.”

In a health expertise discourse, subject positions such as healthy, sick, patient and expert are constructed. The expert subject positions are mainly the positions of doctors, psychologists, nutritionists, physiotherapists, dietary counselors and training experts. For example, in the statement: “Fortunately, the dancers always have someone with them who make sure they get replenished with the right things, such as tailor-made juices and smoothies” you get the impression of the presence of such an expert. A health expertise discourse implies that the experts have the power to set the standards of normality, health and well-being, and the boundaries for when one deviates from the norm. The experts can also encourage the public to follow their advice and recommendations accordingly.

The health experts give advice that the individual is obliged to follow, but can for various reason choose not to. The sick subjects have deviated from an accepted norm, and must rely on expert help to regain health. Thus, they are positioned as subjects lacking power, and partly their autonomy. This is evident in the statement: “A shoulder injury disabled her for two and a half years, something she fortunately got over, thanks to her strong psyche.” Healthy individuals are responsible for their own health and are encouraged to follow the advice of the experts within the discourse. Deviating from expert advice can lead to increased risk of illness. This concords with Markula’s (Markula, 2015) qualitative study on semi-professional dancers’ experiences of injuries. Similar to female athletes, the dancers saw injuries originating from their own lack of body awareness and insufficient understanding of their bodies. If the healthy fails to deal with the expert advice, they can be accused of not knowing what is in their own interest and putting their own health at risk.

A health expertise discourse seems to support science as an institution, which assumes that good health is assessed according to some objective goals. The value neutrality of science rests on an underlying assumption that we can know something about the world that can be revealed through empirical observation. The relationship between the researcher as subject and the research objects is not problematic. Practices within the health care system, such as setting standards for healthy and sick, normal and abnormal, therefore lean heavily on the presumed correct knowledge based on objective, scientific approaches. This demonstrated how these discourses are associated with power. In an expert position one part is entitled to advice someone to lose weight solely based on scientific arguments. Those who possess this body of knowledge are health professionals such as doctors, psychologists, nutritionists, and other professions. Thus, being positioned as an expert within this discourse involves the speaking and taking action from a vantage point.

#### Discourse of Surveillance and Control

The athletic female body constructed as self-control/discipline and dualism seems to be made possible within a discourse of surveillance and control. Self-discipline and the mind’s surveillance of the body seem to presuppose a part of the self which monitors and controls another, seemingly distinct, part. This is illustrated in the quotation “Remember that you can allow yourself a cheat meal a week, especially if this helps you not overeating and keep [“cravings”] in check. Personally, I LOVE tiramisu…”. The use of the word allow may indicate that someone or something is observing our actions and that we are not free to do as we please. In the interviews, the person who monitors and the person being monitored apparently is the same person. This can illustrate the internalization of dominant norms, expectations, and roles in society, which the person strives to conform with. As previously mentioned, such an internalization can be recognized as internalized power by panoptic mechanisms (Duncan, 1994). In an equivalent manner, this is the function of the surveilling part in a discourse of surveillance and control. According to societal norms, individuals may pose a potential threat to themselves and society concerning particular safety parameters such as normal weight, high muscle mass and good health. As a result of self-monitoring through panoptic internalization, the person attempts to comply with what she believes are private standards. This is evident in the quote “I like to know what the food I eat contains (.) I do not like the idea of consuming chemically produced substances, so I always buy organic.” Quotations like “I should perhaps have been stricter with myself when it comes to diet” suggests that the person has an experience of transgressing even though she is not “caught red-handed.”

Transhumanism is also an ideology present in the text material, where an explicit goal is improving humans by the means of modern technology (Harrison and Wolyniak, 2015). Transhumanism therefore involves the assumption that scientifically based claims that improve body, health and lifestyle tantamount morally good knowledge. Transhumanist ideology clearly overlaps with both a discourse of surveillance and control and a neo-liberal discourse: The psyche should have a superior and controlling role over the body’s weaknesses and limitations, and the body has an instrumental value in realizing the person’s goals and ambitions. In particular, this suggests the realization of human potential through scientific innovations. Technological means and its potential become an increasingly naturalized part of daily life, through the use of smartphones and social media to improve fitness (Ferrando, 2013; Meikle, 2016).

#### Discourse of Emancipation

Constructions of the athletic female body as a counterpart to the beauty pressure can be categorized within a discourse of emancipation. Several of the statements describe creating a distance or challenging some established and popular norms concerning how a fit woman should look and what exercise should be all about. This is evident in the quote “It is much more important to have a healthy body than a body that looks good.” The established norms for the female body and exercise are based on the western traditional beauty ideal. The ideal manifests itself as beauty pressure, a strong force that is difficult to identify and resist: “We are probably many who can recognize feeling a certain pressure to exercise. The urge to constantly check numbers and results, and having to control weight and food intake, can quickly become an unhealthy habit. Therefore, Kayla is clear on why she exercises and eats the way she does.”

A discourse of emancipation seems to construct the subject positions of female rebels/liberators and oppressed women. Beauty pressure entails a project in which women are concerned with making themselves more attractive. In order to assess their own attractiveness, women must take an outsider’s perspective and regard themselves through a masculine gaze. The female rebel is actively in opposition to body pressure. The rebel has the ability to point out errors and flaws in the current established standards of the athletic female body. She can also strive to establish new standards and ideals and argue in favor of these new ideals. Thus, the rebels have the legitimacy to encourage others to follow their example and challenge the old assumptions by embracing new ones. In the quote “Are my cellulites showing now? Amanda asks and laughs.” we get the impression that the interviewee ridicules a superficial beauty-focused ideal. By this, the rebels are situated as having authority to declare themselves non-susceptible to the influence of beauty pressure. Thus, they also have the right to regard the oppressed as “victims.” The awareness being potentially influenced by beauty pressure represents a kind of emancipation. When the individual is “liberated,” she has a responsibility to resist the beauty pressure. Detached from this influence she can, to a greater extent, engage in other more fulfilling and rewarding activities, and contribute to improving women’s quality of life. Failure to do so may lead to accusations of being weak and impressionable and having a strained relationship with one’s own body. This is particularly evident in the quote: “In Hollywood, it is impossible to protect yourself from the beauty pressure, but I try to have a healthy relationship to it.” Being positioned as the oppressed, one has limited leeway and opportunities for action. It is not an option to resist nor prevent oneself from being influenced by the beauty pressure, and in most cases, one is not even aware of being under such influence.

The discourse of emancipation, like the neo-liberal discourse, strongly coincides with liberal individualism. In the discourse of emancipation women have a large degree of responsibility in not giving in to the existing beauty pressure and encourage women to go their own way. This complies with Markula’s (Markula, 2001) research on women’s body image distortions, where the individual readers are encouraged by the fitness magazines to take responsibility for their own health and the advice on how to prevent eating disorders. Markula argues that this is strongly guided by a dominant medical understanding of eating disorders, emphasizing the role of the women’s own self-perception in developing a disease by perceiving themselves as obese (Markula, 2001).

In a discourse of emancipation, physical strength, functionality and good health are considered more “valid” ideals for exercise and lifestyle choices. Thus, it enables the subjective experience of exercising for one’s own sake and for the right reasons. Exercise as a beautifying project, on the other hand, is dismissed as evanescent, disadvantageous and oppressive. However, it can be questioned whether a discourse of emancipation really is emancipating. First of all, as previously noted, the physical appearance of the women in the interviews does not really oppose the Western standards of beauty. Secondly, in several ways the discourse seems to represent an apparent settlement with a beauty-focused culture. A discourse of emancipation can be considered as an expression of the fact that one has begun to recognize the adverse effects of slender body ideals, which is illustrated by the research on new forms of eating disorders such as orthorexia (Dunn and Bratman, 2016) and the large number of normal-weighted women who want to lose weight (Ferreira et al., 2016). An emancipating discourse within the athletic female body can also be seen as an example of how women negotiate gender roles in fitness, in line with Rich’s (Rich, 2005) and Markula’s (Markula, 2001) research findings. Although a discourse of emancipation speaks for more collective solutions for body pressure as a social problem, it is nevertheless assumed that one should resist beauty pressure and rather set more appropriate personal goals. For example, a health expertise discourse will focus on getting rid of a vulnerability to be influenced by the “wrong” body ideals. Thus, magazines such as *Xrzise* become channels for expressing the “right” type of body focus. Thus, the discourse of emancipation also entails the concept of healthism: being influenced by the beauty pressure is a matter of making poor decisions. This ignores the systemic societal conditions which has been shown to play important role in the health of the population (Davidson, 2015). In this way, the discourse of emancipation seems to contribute to women’s discontent with their own bodies, rather than counteracting a body focused culture. At best, the emphasis on a more athletic and strong body ideal represents little new. At worst, it is misleading because it is presented as something else entirely; a way for women to free themselves from body pressure to pursue something more empowering.

## Conclusion

Although identified discourses construct the discursive object in different ways, the various constructs’ total effect seems to serve similar purposes. The results from the analysis are related to Duncan’s assumption that inspirational media success stories can have disciplinary effects (Duncan, 1994). Since a neoliberal discourse appeals to the idea of actualizing something already existing within the individual, a project of self-realization depicts the process as a ‘personal struggle’ and can conceal the importance of subtle power structures within our society. Similarly, a health expertise discourse can lead to the invisible disciplinary effects through panoptic mechanisms by offering a legitimate rationale for monitoring the health of the population. However, such effects may be even more difficult to point out when nutritional science and medicine are believed to rest on a value-neutral and empirically based knowledge. The text thus draws heavily on the ideology of healthism, which situated the problem of health and disease at the level of the individual (Crawford, 2006). The discourse of emancipation conveys the impression of disguising the disciplinary effects by undermining beauty pressure and unhealthy body ideals as something women can simply choose not to be influenced by. The analysis also shows that the discourse of emancipation is highly contradictory, constructing superficial body ideals as a thing of the past, and the same time having spokespersons with looks which to a great extent conforms to these “outdated” body ideals.

In the analysis, we have modified Parker’s steps in a Foucauldian discourse analysis. In accordance with Foucauldian discourse analysis being concerned with the construction of subjectivity, we acknowledge that the analysis is regulated and limited by the fact that we, as authors of the text, are situated in specific subject positions too. We consider this as necessary for the analytical process because we assume that we uncover the discursive constructions available to us within our socio-historical context (Parker, 1992).

The decision to analyze interviews rather than journal articles made an impact on the analysis. For example, it is feasible that a neoliberal discourse is particularly evident in texts that profile athletes and other “self-realized” individuals. The use of the term “athletic” in the research question may also be a potential weakness, due to the term not being present in the text material. However, we believe that these decisions are informed by the questions we wanted to answer in the analysis. As the face of the magazine, the interviewees act as role-models the reader can idolize and be inspired by, similar to the role of athletes in society.

One potential and valid objection to our discourse analysis could be the fact that the text material is fairly limited. The inclusion of a greater number of interviews could lead to further nuances of the discursive constructions and possibly several more discourses. This analysis has constituted a good basis for the development of some analytical categories, but call for more research for further elaboration of these discursive tendencies.

### Implications

Exercise, diet, and the body image are topics that constantly appear in the clinical context and in the media. These topics are often communicated with implicit assumptions about the concepts of wellness, health, and fitness. There is also a basic understanding that the individual is able to choose her lifestyle and how she relates to her own body. In continuation of this, phenomena such as eating disorders and preoccupation with food and exercise becomes conditions that affect the individual alone and which are treated accordingly. *Xrzise* as well targets the individual reader when the publisher claims that the magazine is passionate about building a good and healthy self-image for young girls. Identified discourses in the analysis suggests that when it comes to self-esteem and body ideals the magazine ultimately is symptomatic of the problem, rather than a solution. Foucault himself would probably characterize *Xrzise* as a subtle form of disciplining. In this context, the use of a discourse of emancipation in the texts will contribute to obscuring the magazine’s disciplinary function. This becomes even more subtle when the publisher claims that the magazine is a “safe authority” and a credible communicator of healthy values and clear standpoints. Thus, the magazine declares itself as a neutral actor in an expert position when it comes to a specific “truth.”

In a corresponding way to the object of study, *the athletic female body*, we consider the magazine to be part of a complex web of other actors made possible by identified discourses in the analysis, especially a neoliberal discourse. Magazines’ interests are mainly commercial, and as Madsen and Ytre-Arne pointed out, there will be a demand for such magazines and the products they advertise as long as self-realization is potentially limitless project and women fail in achieving this goal (Madsen and Ytre-Arne, 2012). In this sense, *Xrzise* is recognized as the materialization of certain power structures, rather than an independent causative factor.

This analysis is an important contribution to an otherwise one-sided focus of knowledge on a very topical issue. Our analysis has shown that the ostensibly more valid and healthier body ideal, the athletic female body, hardly has any different effects from its skinnier antecedents. The results indicate that constructions of the athletic female body do not rest on “fit” and “health” as neutral concepts. Rather, they are associated with institutional guidelines, power structures and ideology in our socio-historical context. More specifically, we have argued that the text has limiting effects on young women, and may contribute to the maintenance of women’s objectifying relation to their own bodies. *Xrzise* and similar texts contribute to an individualized understanding of body pressure and eating disorders, which causes women to accuse themselves of having a strained relationship with their own bodies rather than focusing on social, cultural and historical^1^ conditions that determine these phenomena.

## Data Availability Statement

The datasets generated for this study are available on request to the corresponding author.

## Author Contributions

The idea of the manuscript was developed in collaboration. SA and RN led roles in the analysis and writing of the manuscript. HB took part in every aspect of the process from analysis to the final draft. All authors contributed to the article and approved the submitted version.

## Conflict of Interest

The authors declare that the research was conducted in the absence of any commercial or financial relationships that could be construed as a potential conflict of interest.
