# Supplementary material for: The Re-shaping of Bodies: A Discourse Analysis of Feminine Athleticism
Source: Front Psychol. 2020 Jul 24;11:1751. doi: 10.3389/fpsyg.2020.01751 (PMC7394218; doi:10.3389/fpsyg.2020.01751)
Supplement: Supplementary file 1 [file Table_1.DOCX]

**Appendix A**

Modified version of Ian Parkers (1992) analytical steps to discourse analysis

| Criterias                                                                         Steps | |
| --- | --- |
| 1. A discourse is realised in texts | 1.     Exploring our object of study through a textual materialization  2.     Exploring connotations through some sort of free association, which is best done with other people |
| 1. A discourse is about objects | 3.     Asking what objects are referred to, and describing them  4.     Analyze the discourse as an object |
| 1. A discourse contains subjects | 5.     Specify what type of subject positions are constructed by the discourse  6.     Analyze the possibilities and limitations for action within a subject position |
| 1. A discourse is a coherent system of statements | 7.     Mapping a picture of the world this discourse presents  8.     Explore how someone would deal with objections to the practices related to the discourse |
| 1. A discourse refers to other discourses | 9.     Setting discourses against each other and explore how adjoining discourses speak about the same objects  10.  Identify points where they overlap, where they constitute what look like same object in different ways |
| 1. A discourse reflects on its own way of speaking | 11.  Referring to other texts to elaborate the discourse and its implicit content  12.  Reflecting on the term used to describe the discourse, a matter which involves moral/political choices on the part of the analyst |
| 1. A discourse is historically located | 13.  Looking at how and where the discourses emerged  14.  Describe how they have changed, and how historical changes are treated as a subject-centered narrative, tending to refer to things which were always there to be discovered |
| 1. Discourses and institutions are mutually supportive | 15.  Identifying institutions which are reinforced when a discourse is used  16.  Identifying institutions that are attacked or subverted when a discourse is used |
| 1. Discourses reproduce power relations | 17.  Looking at which categories of person gain and lose from the employment of the discourse  18.  Looking at who would want to promote and who would want to dissolve the discourse |
| 1. Discourses have ideological effects | 19.  Showing how a discourse connects with other discourses which sanction oppression  20.  Showing how the discourses allow dominant groups to tell their narratives about the past in order to justify the present, and prevent those who subjugated discourses from making history |
